# Systematic identification of single transcription factor perturbations that drive cellular and tissue rejuvenation

**DOI:** 10.1073/pnas.2515183123

**Published:** 2026-01-09

**Authors:** Janine Sengstack, Jiashun Zheng, Turan Aghayev, Gregor Bieri, Michael Mobaraki, Jue Lin, Changhui Deng, Saul A. Villeda, Hao Li

**Affiliations:** ^a^Department of Biochemistry and Biophysics, University of California, San Francisco, CA 94143; ^b^Tetrad Graduate Program, University of California, San Francisco, CA 94143; ^c^Department of Anatomy, University of California, San Francisco, CA 94143; ^d^Developmental and Stem Cell Biology Program, University of California, San Francisco, CA 94143; ^e^Bakar Aging Research Institute, University of California, San Francisco, CA 94143; ^f^Biohub, San Francisco, CA 94158

**Keywords:** rejuvenation, replicative aging, Perturb-seq screening, liver aging

## Abstract

Cellular rejuvenation through transcriptional reprogramming is an exciting approach to counter aging. Using a fibroblast-based model of human cell aging and Perturb-seq screening, we developed a systematic approach to identify single transcription factor (TF) perturbations that promote rejuvenation without dedifferentiation. Overexpressing E2F3 or EZH2, and repressing STAT3 or ZFX, reversed cellular hallmarks of aging—increasing proliferation, proteostasis, and mitochondrial activity, while decreasing senescence. EZH2 overexpression in vivo rejuvenated livers in aged mice, reversing aging-associated gene expression profiles, decreasing steatosis and fibrosis, and improving glucose tolerance. Mechanistically, single TF perturbations led to convergent downstream transcriptional programs conserved in different aging and rejuvenation models. These results suggest a shared set of molecular requirements for cellular and tissue rejuvenation across species.

## Significance

Cellular rejuvenation through transcriptional reprogramming has emerged as exciting approach to counter aging. However, to date, only a few of rejuvenating transcription factor (TF) perturbations have been identified. In this work, we developed a discovery platform to systematically identify single TF perturbations that drive cellular and tissue rejuvenation. Using a classical model of human fibroblast aging, we identified more than a dozen candidate TF perturbations and validated four of them (E2F3, EZH2, STAT3, ZFX) through cellular/molecular phenotyping. At the tissue level, we demonstrate that overexpression of EZH2 alone is sufficient to rejuvenate the liver in aged mice, significantly reducing fibrosis and steatosis, and improving glucose tolerance. Our work expanded the list of candidate rejuvenating TFs for future translation.

Cellular rejuvenation through transcriptional reprogramming is an exciting approach to counter aging and bring cells back to a healthy state. In both cell and animal aging models, there has been significant recent progress in rejuvenation research. Systemic factors identified in young blood through models such as heterochronic parabiosis (in which the circulatory systems of a young and aged animal are joined) rejuvenate various peripheral tissues and cognitive function in the brain (1–4). Partial reprogramming at the cellular level with the Yamanaka factors (four stem cell transcription factors) reverses cellular and tissue-level aging markers and can extend lifespan in old mice (5–8). These discoveries support the notion that transcriptional reprogramming is a powerful approach to improving the health of cells and tissues, and one day could be used as an approach for human therapeutics. However, to date, only a couple of rejuvenating transcription factor (TF) perturbations have been identified (9, 10) and most of them require the overexpression of TFs.

We hypothesized that there are multiple other TF perturbations which could reset cells and tissues back to a healthier or younger state—rejuvenating them. Identifying complementary rejuvenating strategies is important as it will increase the chance of successful future translation. We developed a high-throughput platform, the Transcriptional Rejuvenation Discovery Platform (TRDP), which combines computational analysis of TF binding motifs and target predictions (*Materials and Methods*), global gene expression data of old and young cell states, and experimental genetic perturbations to identify which TF can restore overall gene expression and cell phenotypes to a younger, healthier state. We developed TRDP to be applicable to any cell type, and in both aging and disease settings, with the only requirements being baseline comparison of gene expression data comparing the older/diseased state to the younger/healthier state and the ability to perform genetic perturbations. To model aging in vitro as a validation of our approach, we used the canonical aging model of passaged fibroblasts (11, 12). We tested 400 TF perturbations via our screen and validated reversal of key cellular aging hallmarks in late passage human fibroblasts for four top TFs: E2F3, EZH2, STAT3, and ZFX. Moreover, EZH2 overexpression in vivo rejuvenated livers in aged mice—reversing aging-associated global gene expression profiles, significantly reducing steatosis and fibrosis, and improving glucose tolerance. These findings point to a conserved set of molecular requirements for cellular and tissue rejuvenation.

## Results

### Development of the Transcriptional Rejuvenation Discovery Platform (TRDP).

**Fig. 1.**
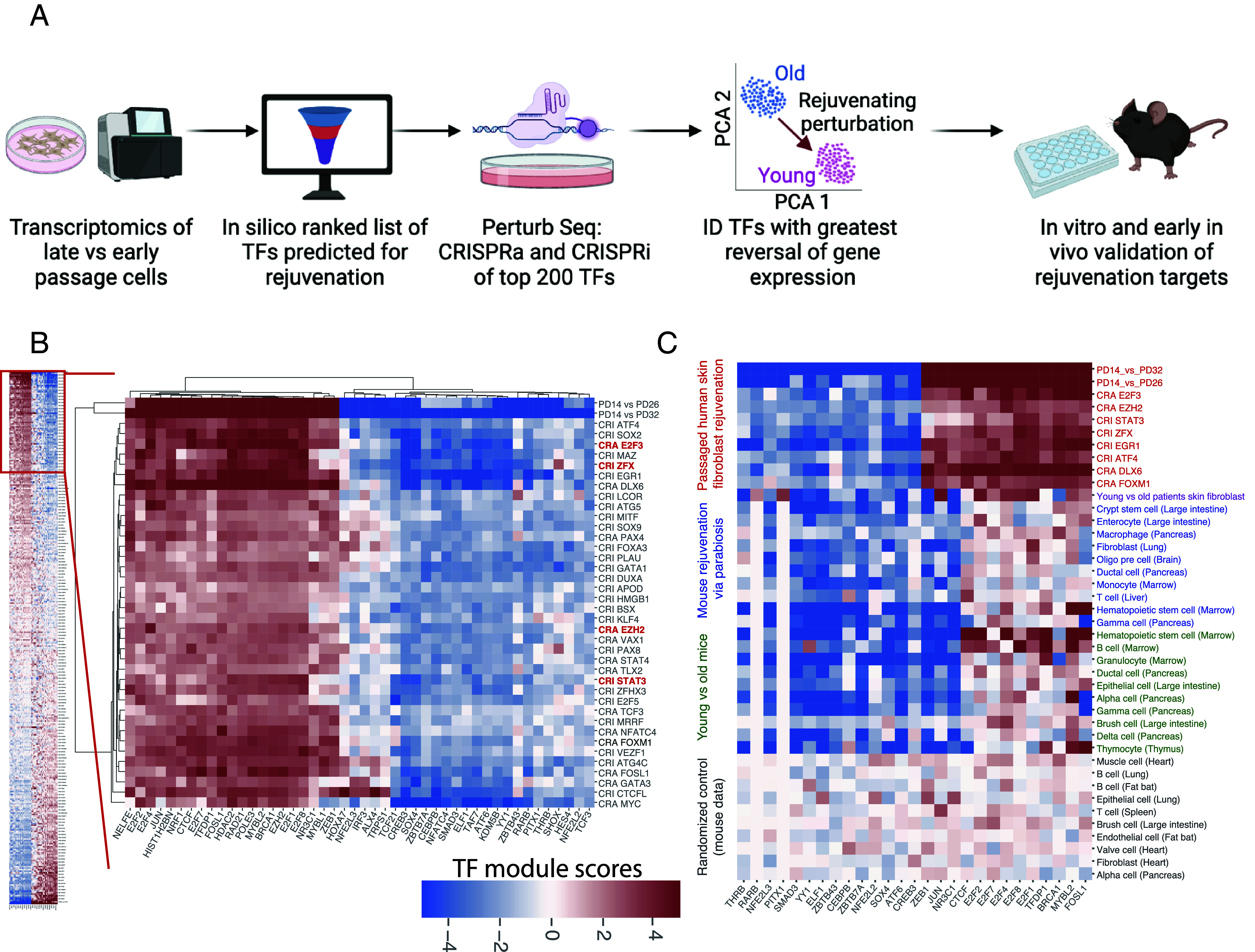
Identification of multiple transcription factor (TF) perturbations that rejuvenate late passage human fibroblasts using the Transcriptional Rejuvenation Discovery Platform (TRDP). (*A*) A flow diagram of TRDP (made using Biorender). Bioinformatic analysis of the transcriptome data of two different states is used to prioritize a list of potential rejuvenating TFs. These TFs are then subjected to Perturb-seq screening, and top hits further validated via. in vitro and in vivo experiments. (*B*) TF module analysis revealed that potential rejuvenating TF perturbations drive similar downstream gene expression changes. TF perturbations (rows) were clustered by the TF module scores (indicated by the color of the pixels) for selected TF modules (columns) from the SCENIC analysis. The TF module score is derived from the comparison of the AUCell scores between two groups of cells (such as CRA_E2F3 vs. CRA_NT) (*Materials and Methods*). Only the modules differentially expressed between WT PD 14 (early passage) and PD 32 (late passage) (|TF module score| > 5) were shown. (*C*) Transcriptional signature defined by rejuvenating TF perturbations was shared across species and cell types in different models of aging and rejuvenation. Shown are TF module scores comparing different pairs of cells (rows) organized into 5 colored blocks: 1) early passage and late passage human fibroblasts, and selected rejuvenating TF perturbations and their NT controls (red); 2) skin fibroblasts from young and old patients (purple, data from Ref. 15); 3) mice rejuvenated via parabiosis (aged mice from the aged-young pairs) and control mice (aged mice from aged–aged pair), for the tissues/cell types indicated (blue, data from Ref. 16); 4) young mice (from the young–young pair) and old mice (from the old–old pair) (green, data from Ref. 16); 5) randomized controls (black), where for a given tissue/cell type, the two groups of cells were merged and randomly repartitioned into two groups of the same sizes. For blocks 3 to 5, only the top 10 hits (those most similar to PD14 vs. PD32 profile) were selected.

Our underlying hypothesis is that perturbing a single TF can lead to downstream changes which shift a cell state from old/diseased back towards young/healthy. To develop our platform and test this approach, we utilized the classic cell aging model—passaged human neonatal dermal skin fibroblasts—to measure hallmarks of cellular replicative aging, wherein early, middle, and late passages reflect some aspects of young, middle-aged, and old cells (12). The first step of TRDP (Fig. 1*A*) is to perform global transcriptomics of the two cell states. Then, bioinformatics tools (*Materials and Methods*) are used to predict the genes regulated by each TF based on a combination of known TF binding motifs and their occurrence proximal to the promoters of each gene. TRDP compiles a list of TFs’ target genes and identifies which were most significantly differentially expressed between the cell states. From this, TRDP outputs a ranked list of TFs which likely regulate the most gene expression differences between the two cell states. Next, we follow up on these top TF perturbations in vitro and in vivo with single cell RNA sequencing and phenotyping. To systematically perturb TFs in vitro, we used Perturb-seq (13, 14), which uses CRISPRa (CRA) for activation of a target gene or CRISPRi (CRI) for inhibition of a target gene in cells followed by single-cell RNA sequencing. Then, for further validation of these genes in vitro and in vivo, other tools can be used for genetic perturbations, such as an AAV virus delivery of an overexpression plasmid for in vivo expression. The final output of TRDP is a TF perturbation, or collection of perturbations, which lead to rejuvenation of an older cell or tissue back towards a younger state, as defined by gene expression and cellular phenotypes.

**Fig. 2.**
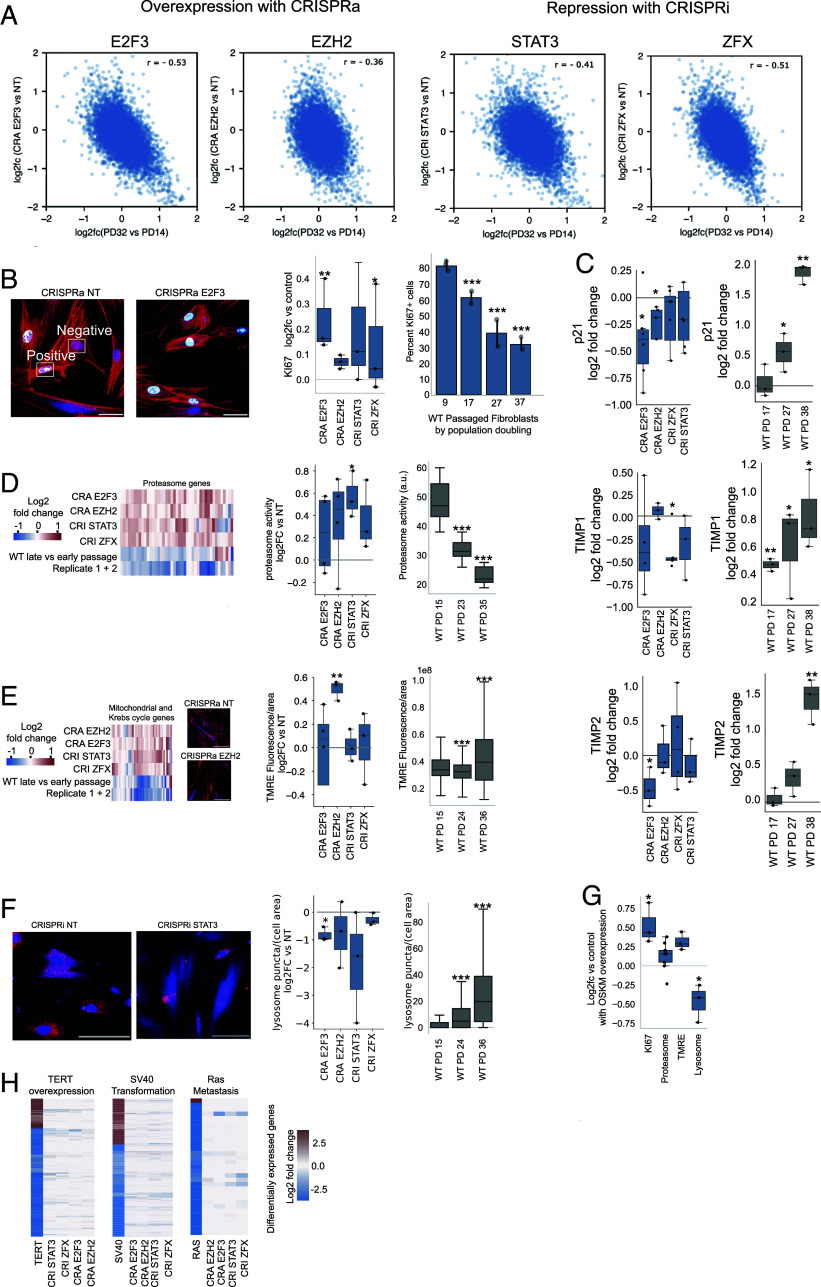
Overexpressing E2F3 or EZH2, or repressing STAT3 or ZFX led to the reversal of classic hallmarks of aging back toward an earlier passage stage. (*A*) Correlation plots comparing gene expression changes [log2(fold change)] between late passage and early passage WT cells to that between late passage cells with a TF perturbation and those with the NT control. Perturbations with a significant negative correlation (R_rej) indicated that the TF perturbation reversed global gene expression changes back toward an early passage stage. (*B*) KI67+ microscopy and quantification of CRISPRa, CRISPRi, and WT passaged fibroblasts. Blue is Hoechst staining the nucleus, red is phalloidin staining actin, and green is KI67. (*C*) Quantitative PCR (qPCR) measurements of p21 (CDKN1A), TIMP1, and TIMP2; log2 fold change in CRISPRa and CRISPRi is relative to non-targeting (NT) treated controls and log2 fold change in WT is relative to PD 9 fibroblasts. (*D*) Proteasome gene expression and activity. (*E*) Mitochondrial and Krebs cycle gene expression and TMRE membrane potential staining. (*F*) Lysosome staining and quantification. (*G*) Percent changes in KI67+, proteasome activity, mitochondrial TMRE staining, and lysosome staining in late passage fibroblasts overexpressing the Yamanaka factors (OCT4, SOX2, KLF2, and MYC). 50 μM Scale bar on all microscopy, *P < 0.05, ** *P* < 0.01, *** *P* < 0.001 (see *Materials and Methods* for the statistical analysis). (*H*) Differentially expressed genes found in human skin fibroblasts progressively being turned cancerous and the expression of those genes under our TF perturbations. For TF perturbations, log fold changes between the perturbation and nontargeting control were shown. For natural passaging data with multiple points, sometimes (e.g., percent of MKI67) the absolute values instead of fold changes were shown.

Through the computational pipeline of TRDP, we selected the top 200 candidate TFs to perturb in late passage fibroblasts, to try identifying the perturbations that reverse the gene expression in late passaged cells to that of early passaged cells (list of TFs in Dataset S1). Based on phenotyping we performed (data in Fig. 2), early passage cells were 0 to 20 population doublings (PD), middle passage cells were 21 to 30 PD, and late passage cells were over 31 PDs. We performed Perturb-seq screening of these top 200 TFs using CRISPRa and CRISPRi, followed by differential gene expression analysis in comparison to that of the baseline states (early vs late passage). Nontargeting sgRNA were used as controls and are noted as either CRA NT (for CRISPRa cell line with nontargeting sgRNA) or CRI NT (for CRISPRi cell line with nontargeting sgRNA). We ranked TF perturbations by those with the greatest global reversal of gene expression toward early passage cell state. We quantify the degree of reversal by R_rej, defined as the correlation between log fold change of gene expression of late passage cells vs. early passage cells and log fold change of late passage cells with a TF perturbation vs. late passage cells with a nontargeting control. TF perturbations with a significant negative R_rej indicated the perturbation reversed the gene expression changes due to replicative aging. We then ranked R_rej and selected the top hits (TFs with r =< −0.3 Table 1).

**Table 1.** Transcription factor (TF) perturbations led to significant reversal of global gene expression back to an earlier passage state

CRA		CRI	
gene	R_rej value	gene	R_rej value
DLX6	−0.57	EGR1	−0.55
**E2F3**	−0.53	**ZFX**	−0.51
FOXM1	−0.47	ATF4	−0.48
FOSL1	−0.45	VEZF1	−0.47
NFATC4	−0.41	MAZ	−0.47
MYC	−0.4	SOX2	−0.47
STAT4	−0.39	PAX8	−0.44
GATA3	−0.38	ZFHX3	−0.43
**EZH2**	−0.36	ATG4C	−0.42
PLAU	−0.35	PLAU	−0.41
HSF2	−0.35	ATG5	−0.41
PAX4	−0.33	HMGB1	−0.41
NKX2-2	−0.32	**STAT3**	−0.41
SIM2	−0.32	GATA2	−0.4
VAX1	−0.3	KLF4	−0.39

To have a global view of the downstream transcriptional response to the TF perturbations, we performed TF module analysis using the Scenic algorithm (17). TF modules are defined as groups of genes regulated by a TF, based on their covariation with the TF across single cells and the occurrence of the TF binding motif in their promoters (17) (*Materials and Methods*). We found that the top hit TF perturbations resulted in similar downstream gene expression changes at the level of TF modules, when viewed from a subset of the modules differentially expressed between late passage (PD32) and early passage (PD14) cells (Fig. 1*B*), even though the factors themselves originated from diverse upstream pathways. We observed similarities in the gene expression changes in these top TF perturbations as in other aging and rejuvenation models, including in young patients’ human skin fibroblasts (15), various tissues from young mice (e.g., intestine, pancreas, marrow, and thymus) (16), and various tissues from old mice rejuvenated through heterochronic parabiosis (e.g., intestine, pancreas, marrow, lung, liver, and brain) (16) (Fig. 1*C*). Cell types in rejuvenated aged mice that exhibit this transcriptional signature are enriched for mitotically active cells, such as large intestine crypt stem cells, pancreas ductal cells, lung fibroblasts, and hematopoietic stem cells. These observations suggest that there could be a common set of gene expression changes, perhaps of proliferative cells, across species, cell types, and different rejuvenation methods.

It is clear that a major component of the downstream gene expression program is related to cell cycle, i.e., most of the top TF perturbations up-regulate cell cycle–related TF modules (e.g. several E2F family TF modules (Fig. 1*B* and *SI Appendix*, Fig. S1), and thus should boost cell cycle progression. This is confirmed by classifying single cells into different cell cycle phases and observing that most of the top TF perturbations decrease the percent of cells in G1 phase while increasing the percentage in S and G2/M phases (*SI Appendix*, Fig. S2). However, many top TF perturbations also reverse gene expression changes between late passaged and early passaged cells not obviously attributable to cell cycle redistribution, as shown by the R_rej calculated when restricted to a subset of cells in the same cell cycle phase (Dataset S2). E.g., when comparing the changes between late passage and early passage cells in G1 phase with those between TF perturbed cells and NT control cells in G1 phase, several top hit TF perturbations still have a significant R_rej. One example is EZH2 overexpression. Genes down regulated in late passage cells in G1 phase but reversed with EZH2 overexpression include a broad range of classes such as RNA processing, one carbon metabolism, protein synthesis, mitochondrial function, and stress response (Dataset S3).

Gene expression patterns at transcription module level are largely similar among the top hit TF perturbations when restricted to TF modules differentially expressed between early and late passaged cells, which is to be expected as we selected TF perturbations based on their similarity to early passage/late passage comparison. However, different TF perturbations do cause distinct gene expression patterns when viewed from all the TF modules generated from the single-cell data (17) (*SI Appendix*, Fig. S1). Some of these gene groups are unique to a given TF perturbation, indicating potential specific downstream phenotypes that may differentiate different TF perturbations.

We checked whether CRISPRa and CRISPRi of the same TF produced opposite gene expression changes, and we found that very often this is not the case (Dataset S4). While for some TFs (such as EZH2 and EGR1) overexpression and repression did lead to opposite global gene expression changes, for the majority of TFs there is no concordance. We speculate that some of these TFs are necessary but not sufficient for the expression of the target genes, while others may be sufficient but not necessary. Thus, perturbation in one direction may produce effect but not in the opposite direction (e.g., if a TF is sufficient but not necessary, overexpression will produce an effect while repression may not produce any effect).

### Top TF Perturbations Identified By TRDP Reversed Several Cellular Aging Hallmarks in Passaged Fibroblasts.

To validate the top hits from the Perturb-seq screen, we performed comprehensive cellular and molecular phenotyping of various aging hallmarks (18) in late passage fibroblasts with a single TF targeted. Specifically, we focused cellular studies on four TF perturbations that reversed overall gene expression back to an earlier passage state: overexpression of EZH2 or E2F3 and repression of STAT3 or ZFX. E2F3 is largely involved in regulating cell cycle progression from G1 to S phase (19). EZH2 is a methyl-transferase best known for being a catalytic subunit of the polycomb repressive complex 2 (PRC2) (20) but has other roles outside the PRC2 complex (21–24). STAT3 is a member of the STAT family that forms part of the JAK-STAT signaling cascade and plays an important function in immune/inflammatory response (25). ZFX is a relatively poorly understood TF (26), but it has links to stem cell renewal (27, 28). We selected these four single TF perturbations initially because they had a large effect on reversing aging gene expression, as seen from R_rej (Fig. 2*A*), and from the shift of the single cell distribution in the gene expression space towards early passage cells (*SI Appendix*, Fig. S3). They also came from distinct pathways and were relatively unexplored in terms of rejuvenation research.

We assayed a wide range of cell aging phenotypes, including proliferation, proteostasis, and mitochondrial function. Perturbations of any of these four factors in late passage fibroblasts led to slightly higher rates of cellular proliferation, ranging from 10 to 30% more on average, and decreased senescence-related gene expression (Fig. 2 *B* and *C*), bringing both back towards middle passage levels. These single TF perturbations also improved cellular proteostasis. Both proteasome gene expression and proteasome activity returned to a middle passage state, with up to ~40% increases (Fig. 2*D*). Mitochondrial gene expression and activity, both of which decrease with age and senescence, were significantly increased (Fig. 2*E*). The total lysosome staining decreased, once again returning to a middle passage state (Fig. 2*F*). Previous literature shows that the lysosomes of late passage cells gradually transform to residual bodies (containing undigested materials), and these bodies increase in number and size, reflecting degeneration of lysosomal function observed at older age (29, 30). As a comparison to these factors, we performed overexpression experiments of the Yamanaka factors (31) in late passage fibroblasts and validated increased mRNA expression by qPCR (*SI Appendix*, Fig. S4); note that OCT4 expression was too low in control cells to accurately measure changes in expression. The fold change caused by overexpressing the Yamanaka factors in cell proliferation, proteasome activity, mitochondria activity, and lysosome were comparable to those seen with our four single TF perturbations (Fig. 2*G*). Thus, single TF perturbations we identified led to amelioration of some replicative and cellular aging hallmarks comparable to the combined effect of the Yamanaka factors in an in vitro context.

These single TF perturbations did not generally cause significant changes in DNA damage, telomere length, methylation age, nor fibroblast identity related genes (*SI Appendix*, Figs. S5 and S6). The observation that the methylation age did not change with the TF perturbations is consistent with the previous report that cell senescence is unrelated to epigenetic clocks (32). We also found that the transcriptomic responses to the TF perturbations have no resemblance to those caused by cancerous transformations (Fig. 2*H*), arguing that these TF perturbations are not tumorigenic. These results indicate that these factors may target orthogonal pathways to factors related to stem cells or dedifferentiation. Thus, through TRDP, we identified TF perturbations which can each individually reverse aspects of passaged dermal fibroblast aging.

We note that there are some specificities in terms of phenotypes reversed by each TF perturbation. For example, E2F3 overexpression has the strongest effect on increasing proliferation (percent of KI67 positive cells, Fig. 2*B*), EZH2 overexpression is best at increasing mitochondria activity (Fig. 2*E*), while repression of STAT3 produced the strongest effect on increasing proteasome activity and lysosome function (Fig. 2 *D* and *F*). These differences are possibly caused by differences in the downstream transcriptional programs driven by the TF perturbations.

### Early In Vivo Proof of Concept Data Shows 3-wk Liver-specific Overexpression of EZH2 in Aged Mice Livers Reversed About 8 mo’ Worth of Aging-Associated Global Gene Expression, Steatosis, Fibrosis, and Impaired Glucose Tolerance.

**Fig. 3.**
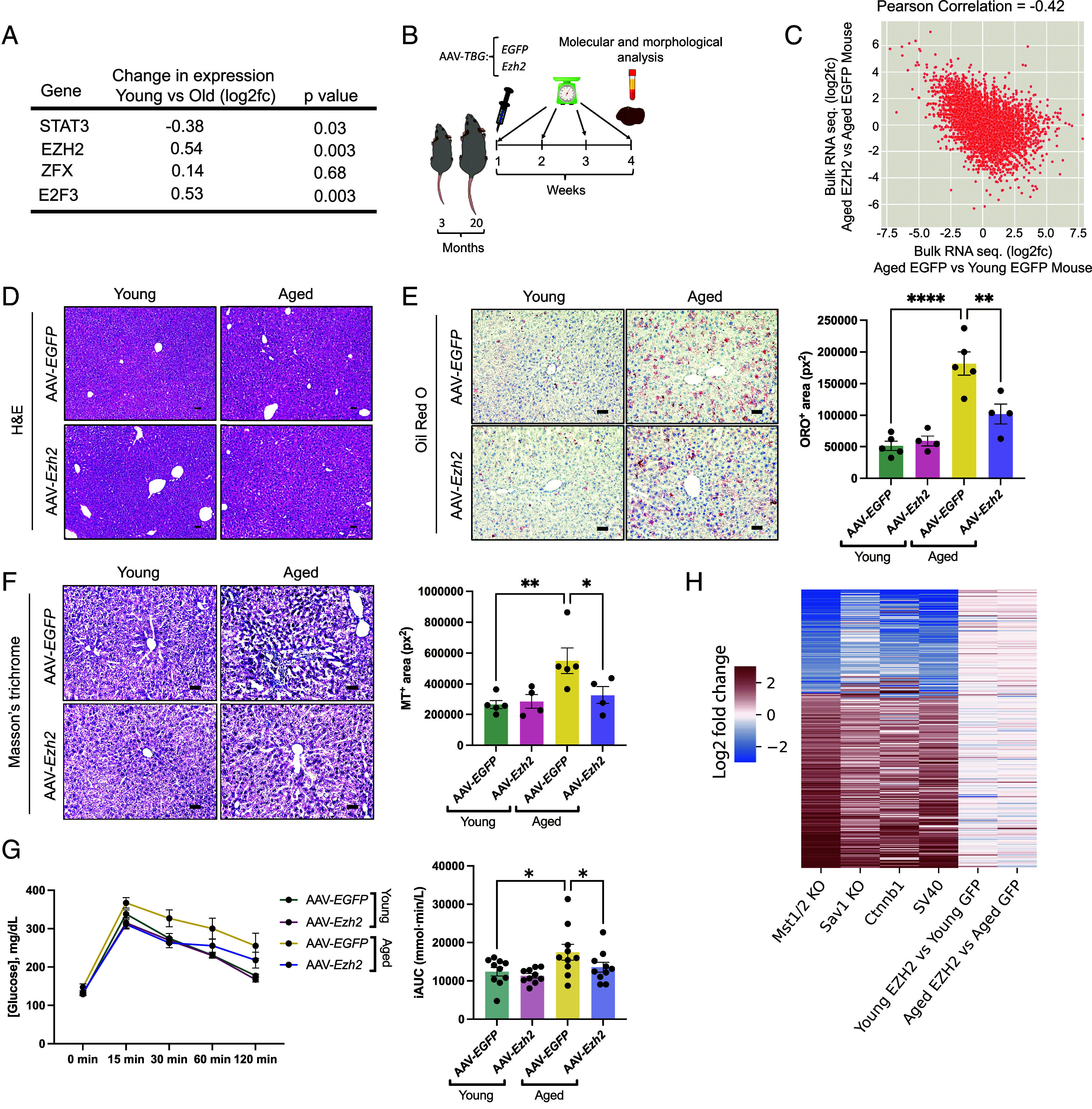
EZH2 overexpression in aged mouse livers reversed global gene expression, steatosis, fibrosis, and impaired glucose tolerance back toward a young mouse phenotype. (*A*) Gene expression changes of our top four TF in aged versus young mice, as measured through bulk RNA sequencing. (*B*) Schematic of the experiment. (*C*) Correlation plot of the liver bulk RNA sequencing comparing aged control mice (n = 3) versus young mice (n = 4) on the x-axis and aged EZH2 overexpression mice (n = 4) versus aged control mice on the y-axis. For each group, gene expression data are averaged across individual mice. (*D*) Representative images of Hematoxylin and Eosin (H&E) stain of the liver sections of young and aged mice infected with either AAV-*EGFP* or AAV-*Ezh2* after three weeks of treatment. (*E*) Representative images and quantification of Oil Red O stain of the liver sections of young and aged mice infected with either AAV-*EGFP* or AAV-*Ezh2* (n = 4 to 5 per group) (*F*) Representative images and quantification of Masson’s trichrome stain of the liver sections of young and aged mice infected with either AAV-*EGFP* or AAV-*Ezh2* (n = 4 to 5 per group). Data are mean ± SEM. *P < 0.05, **P < 0.01 ****P < 0.0001 (one-way ANOVA with Šídák’s correction for multiple comparison). (*G*) Glucose tolerance tests (GTT) were performed at week 3 after an overnight fast. Blood glucose was measured at the indicated time points following intraperitoneal glucose administration (*Left* panel). Glucose excursion curves and incremental AUC quantification for young and aged mice infected with AAV-EGFP or AAV-Ezh2 (n = 10 mice/group). Data are mean ± SEM. *P <= 0.05, one-way ANOVA with Newman–Keuls correction for multiple comparisons. (*H*) Gene expression profiles of mouse liver cancer models (mst1/2 knock out, Sav1 knock out, activated Ctnnb1, and SV40 T antigene transgenic) compared to transcriptional response to EZH2 overexpression in mouse liver (young and old). Genes are selected based on differential expression in the cancer models (|log2fc| > 1 in at least three models). There are a set of consistently up-regulated genes and a set of consistently down-regulated genes across the models. Most of these genes are not significantly altered in response to EZH2 overexpression.

Thus far, we used TRDP to predict TF perturbations which could reverse gene expression—and some aspects of cellular aging—in passaged fibroblasts. Next, we asked whether a single TF perturbation validated in vitro could lead to reversal of aging phenotypes at the tissue level in vivo. We focused on the aged liver as it is both responsive to rejuvenation interventions, such as heterochronic parabiosis (1, 33), and amenable to temporally controlled and tissue-specific gene expression manipulations (34). We first analyzed bulk RNA sequencing data of young (3 mo) and aged (20 mo) mouse livers and looked at whether there were significant age-related differences in the expression of E2F3, EZH2, STAT3, and ZFX. EZH2 and E2F3 expression were significantly higher in young mice, STAT3 was significantly higher in old mice, and ZFX was not different (Fig. 3*A*). We elected to further investigate EZH2 given its relative better safety profile compared to E2F3 (35, 36) and fewer previous links to liver health unlike STAT3 (37).

We used a liver-specific viral-mediated overexpression approach, where young and aged mice were administered AAV8 encoding EZH2 or a control vector (EGFP control) via tail vein injection. Changes in gene expression and liver histopathology was assessed three weeks post injection (Fig. 3*B*). EZH2 was successfully overexpressed compared to control (log2fc = 2.9, or ~650% more), which resulted in the expression of thousands of genes being reversed back toward the young mouse state (Fig. 3*C*). The degree of correlation between aged/young and aged EZH2/aged control (R_rej = -0.42) is even stronger than that observed from the in vitro screening in human fibroblasts (R_rej = −0.36). We found that genes down-regulated in aged mouse livers and reversed by EZH2 overexpression (|log2fc| > 1.5 in both old/young and EZH_OE/control comparisons) encompassed a broad range of functions with enrichment in “transmembrane transporter” category. Genes up-regulated in aged mouse livers and reversed by EZH2 overexpression were enriched for “leukocyte mediated immunity” and “cardiac muscle cell action potential” (Dataset S5). These data indicate that EZH2 overexpression reversed age-related inflammation/immune response, loss of cell identify (possibly through epigenetic drift), and loss of transporter genes important for liver function.

EZH2 overexpression did not change general liver morphology within this 3-week period nor lead to any histological aberrations (Fig. 3*D*). Importantly, EZH2 overexpression did reverse hallmarks of liver health back to a young mouse state, with both a decrease in steatosis (Fig. 3*E*) and fibrosis (Fig. 3*F*) to at least half the level of the old mouse livers. Gene expression changes are concordant with rejuvenated histological phenotypes. For example, expression of lipid metabolism–related genes returned back to levels indicative of a more youthful state following EZH2 overexpression (*SI Appendix*, Fig. S7). Glucose tolerance tests (GTT) revealed that EZH2 overexpression in aged mice restored systemic glucose metabolism, as reflected by improved glucose clearance toward levels observed in young mice (Fig. 3*G*). EZH2 treatment also caused a decrease in inflammatory proteins, p-SAPK/JNK, but no change in p16 (*SI Appendix*, Fig. S8 *A*–*D*). Liver damage was not detected, observing no change in ALT activity as well as no differences in body weight (Fig. S8 *E* and *F*). Together, these data indicate that a TF perturbation like EZH2 overexpression identified by TRDP has the potential to be beneficial in vivo.

To assess potential cancer risk, we compared the liver transcriptome of aged mice with EZH2 overexpression with that from mouse liver cancer models. We analyzed several published datasets from independent experiments in which different treatments were used to cause liver cancer in mice (38). Our analysis indicates EZH2 overexpression did not share a gene expression signature with cancerous gene expression (Fig. 3*H*). Among the cancer datasets, there were clear conserved gene expression signatures, including up-regulation of genes related to stress response, cell adhesion/migration, regulation of cell population proliferation, and blood vessel development, and down-regulation of carboxylic acid metabolism and amino acid metabolism genes (Dataset S6). These signatures suggest a set of changes that are necessary for the growth/migration and metabolic rewiring of the cancer cells. EZH2 overexpression mouse liver profiles have no resemblance to these gene expression signatures. This, together with the histology and functional data, argues that EZH2 overexpression is not simply pro-oncogenic.

## Discussion

In summary, we developed a platform, the Transcriptional Rejuvenation Discovery Platform (TRDP), which combines bioinformatics knowledge of TF binding motifs and target predictions (*Materials and Methods*), global gene expression data of old and young cell states, and genetic perturbations to identify which TF can restore overall gene expression and cell phenotypes to a younger, healthier state. We performed early validation of this approach in vitro with passaged fibroblasts and in vivo, with EZH2 short-term overexpression. In vitro, we provide gene expression and functional evidence that the individual perturbation of E2F3, EZH2, STAT3, and ZFX can reverse some of the classic replicative aging and cellular aging hallmarks—with some factors increasing proliferation, proteostasis, or mitochondrial activity. In our early-stage in vivo work, we demonstrate that overexpression of EZH2 is sufficient to reverse aspects of liver aging in old mice. These results support the utility of TRDP as a method for identifying and testing novel TF perturbations for rejuvenation phenotypes. Mechanistically, we also observed that perturbing TFs with a diverse range of function can elicit similar downstream transcriptional programs and cell phenotypes.

We surmised that these TF perturbations reversed aging hallmarks, rather than slowing down the aging process, because the effects generated by these perturbations are much larger within the time frame of the experiment than what we would expect from slowing down aging. On average, the TF perturbations caused late passage cells to have cellular phenotypes more like middle passage cells. This change occurred within about three population doublings (duration of the experiment), while the cells’ phenotypes reset back the equivalent of 12 to 14 population doublings. In the early in vivo work with EZH2 overexpression, just three weeks of overexpression led to approximately a 50% reversal in gene expression, steatosis, fibrosis, and impaired glucose tolerance, which had accumulated over an entire lifetime (~20 mo).

A big challenge facing the rejuvenation field is to ensure that rejuvenating genetic manipulations do not lead to increase in cancer incidence. Given that we used a replicative aging model to perform the screen, it is a natural concern that the TF perturbations that reverse replicative aging (and thus increase cell proliferation) may be protumorigenic (39, 40). We have shown that in the human fibroblast model, the transcriptome responses to the TF perturbations have no resemblance to those caused by oncogenic transformations (Fig. 2*H*). Furthermore, these TF perturbation partially reverse “mesenchymal drift” (MD), recently identified as a conserved gene expression signature of aging across tissues (41) (*SI Appendix*, Fig. S9). We also found that the transcriptome response to EZH2 overexpression in the liver of old mice has no similarity to the conserved gene expression signature derived from different mouse liver cancer models (Fig. 3*H*). In addition, EZH2 overexpression in liver significantly improved glucose tolerance of the old mice, while in mouse liver cancer models, glucose tolerance in general is impaired due to misregulated metabolism (42, 43). EZH2 overexpression also reverses fibrosis which is common in liver cancer. Together, this evidence argues that these TF perturbations are not simply protumorigenic; instead, they reverse the aspects of aging that are not directly related to cell proliferation. However, the TF perturbations we performed were relatively short term, e.g., the EZH2 overexpression treatment was for three weeks. Thus, we cannot exclude the possibility that long-term treatment may increase the cancer incidence. Given that EZH2 overexpression in the long term is associated with (although not causally linked to) cancer risk (44), and that EZH2 appears to negatively influence lifespan in some model organisms (45), it is important to analyze the long-term effect of EZH2 overexpression in multiple tissues in future studies. It will also be important to study how different dosages and durations of the treatment will influence the outcome.

We note other limitations of the model we used for the screening, i.e., the replicative aging model of the fibroblast. Although there are many aging hallmarks shared between this in vitro model and in vivo aging (46), there are also important differences. While the gradual slowdown of cell cycle progression is a major hallmark of in vitro aging, this may not be an important feature of in vivo aging and is not relevant to postmitotic cells. Thus, future work is needed to generalize the approach to perform in vivo screening and in different cell models, especially models for post-mitotic cells.

TRDP utilizes global gene expression as a readout, in contrast to a predefined set of markers, which we think is more useful and unbiased when assessing complex phenotypes such as aging where a large number of genes change their expression. While we used TRDP for aging-specific TFs, TRDP can be used as a strategy to tackle other challenging problems, such as identifying TF perturbations to correct a diseased state back to a healthy state. Indeed, our in vitro and in vivo data indicate that single TF perturbations— first predicted by TRDP—can lead to robust and significant changes across wide-ranging pathways linked to cellular aging and disease.

## Materials and Methods

Passaged primary human skin fibroblasts were used to profile the transcriptome of “old” (late passage) and “young” (early passage) cells. A candidate list of 200 TFs was selected based on the differential expression of the TF modules between the old and the young states and literature search. Perturb-seq screening was performed using CRISPR_a (activation) and CRISPR_i (inhibition) lentiviral guide libraries and fibroblast cells with the corresponding CRISPRa and CRISPR_i machines. Validation in cell culture was performed by assaying various aging hallmarks. In vivo validation was performed by overexpressing EZH2 in the liver of young and old mice, followed by gene expression, histology, and functional analyses. For the details of the *Materials and Methods*, see *SI Appendix*.

## Supplementary Material

Appendix 01 (PDF)

Dataset S01 (XLSX)

Dataset S02 (XLSX)

Dataset S03 (XLSX)

Dataset S04 (CSV)

Dataset S05 (XLSX)

Dataset S06 (XLSX)

Dataset S07 (XLSX)

Dataset S08 (XLSX)

## Data Availability

All data needed to understand and assess the conclusions of this study are included in the text, figures, and supplementary materials. The full set of perturb-seq data and mouse liver gene expression data are available from figshare (https://doi.org/10.6084/m9.figshare.30898748, and https://doi.org/10.6084/m9.figshare.30899018) (47, 48).
