# Supplementary material for: Systematic identification of single transcription factor perturbations that drive cellular and tissue rejuvenation
Source: Proc Natl Acad Sci U S A. 2026 Jan 9;123(2):e2515183123. doi: 10.1073/pnas.2515183123 (PMC12799168; doi:10.1073/pnas.2515183123)
Supplement: Supplementary file 1 — Appendix 01 (PDF) [file pnas.2515183123.sapp.pdf]

PNAS

Supporting Information for

Systematic identification of single transcription factor perturbations that drive cellular and tissue rejuvenation

Janine Sengstack[†, 1, 2, 3], Jiashun Zheng[†, 1], Turan Aghayev[†,4], Gregor Bieri[4], Michael Mobaraki[1, 5], Jue Lin[1], Changhui Deng[*, 1], Saul A. Villeda[*, 4,6], Hao Li[*,1,7]

[1]Department of Biochemistry and Biophysics, University of California, San Francisco, San Francisco, CA, USA

[2]Tetrad Graduate Program, University of California, San Francisco, San Francisco, CA, USA

[3]Current address: Junevity, Inc., San Francisco, CA 94107, USA

[4]Department of Anatomy, University of California, San Francisco, San Francisco, CA, USA

[5]Developmental and Stem Cell Biology Program, University of California, San Francisco, San Francisco, CA, USA

[6]Bakar Aging Research Institute, University of California, San Francisco, San Francisco, CA, USA

[7]Biohub, San Francisco, CA 94158, USA

†These authors contributed equally for this work.

*Correspondence: changhui.deng@ucsf.edu, Saul.Villeda@ucsf.edu, haoli@genome.ucsf.edu

This PDF file includes:

Supporting text
Figures S1 to S9
Legends for Datasets S1 to S8
SI References

Other supporting materials for this manuscript include the following:

Datasets S1 to S8

Supporting Information Text

Materials and Methods

Lentivirus production

Lenti-X 293T (Lx293T) were used for lentivirus production. Lx293T cells were grown in Dulbecco's modified eagle medium (DMEM) supplemented with 10 % FBS and penicillin-streptomycin. Lentivirus was made by transfecting Lx293T with standard packaging vectors and TransIT-LT1 Transfection Reagent (Mirus, MIR 2306). Viral supernatant was harvested two days after transfection, filtered through a 0.45 um filter, and either added directly to target cells or frozen in aliquots at -80 °C.

Plasmids

All the CRISPR related plasmids were gifts from the Jonathan Weissman lab (pMH0001, pJKNp44, pJR89, pJR85, pMJ114, pMJ117, and pMJ179). For dual sgRNA production, previous protocols were followed. The final plasmids were validated via sequencing. For Yamanaka factor overexpression, Addgene plasmid #24603 was used.

Cell culture and CRISPRa and CRISPRi cell lines

Neonatal primary skin fibroblasts were purchased from ATCC (PCS-201-010) and cultured in ATCC's Fibroblast growth kit with low serum (PCS-201-041), with phenol red (ATCC, PCS-999-001) and penicillin-streptomycin (ATCC, PCS-999-002). These fibroblasts were passaged for almost one year, during which they were split about 1:2 or 1:4 at about 80 - 90 % confluency. Population doublings were determined using cell counting, by which we compared the number of cells plated initially to the number of cells at the next passage.

Stable cell lines in passaged fibroblasts expressing either CRISPRi (CRI; pMH0001) or CRISPRA (CRA; pJKNp44) were created by infecting either the CRA or CRI lentiviral particles into early passage fibroblasts. These vectors have a BFP tag, and thus we sorted cells for purity by BFP on the BD FACSAria2. CRA and CRI cell lines were passaged until they were late passage; BFP fluorescence and CRA/CRI activity was maintained across all population doublings. Next, sgRNA lentiviral particles were infected into CRA and CRI cells at the desired population doubling at an MOI of ~0.3. For all experiments using sgRNA, CRA or CRI cells were infected with the sgRNA lentiviral particles, recovered for two days, selected for purity using puromycin for 2-3 days (2µg/mL), recovered for an additional 2 days, and then used for experiments. The sgRNA vector had a BFP tag as well, and this one was significantly brighter than the CRA/CRI vectors' BFP. The puromycin selection led to 90 - 100% purity, which we could visualize by the very bright BFP signal from the infected cells.

Real-time quantitative polymerase chain reaction (qPCR)

Total RNA was isolated using the RNeasy Plus Mini Kit (Qiagen, 74134). RNA was converted to cDNA using the SuperScript IV (Invitrogen, 18090050) standard protocol. Twenty uL qPCR reactions were prepared with 10 uL of the KAPA SYBR FAST Universal MasterMix (Roche, KK4602), 5 uL cDNA (representing 5 - 20 ng RNA per reaction), and 5 uL of forward and reverse primers mixed together 1:1 at 0.8 uM each. Three technical replicates were run for every sample. These reactions were run on the LightCycler 480. Relative expression of each gene (ΔCt) was measured using beta-actin as a control gene. Log 2 fold change (log2fc) was calculated by finding the difference between two conditions'ΔCts ($\Delta\Delta$Cts). The primers are listed in Dataset S7.

Single-cell RNA sequencing and Perturb-seq

For our first round of WT passaged cell scRNA-seq, we used manufacturer's protocols for the Chromium Single Cell 3' Library v2 (10x Genomics, 120237) with one change; different WT passage stages were added in a pool and identified with cell membrane barcodes(1). For the second round of WT passage cell scRNA-seq, we used the same set up, except we used the Chromium Next GEM Single Cell 5' Library v1.1 (10x Genomics, 1000167).

For the CRA/CRI Perturb-seq experiment with dual sgRNA, the top two guides for every TF and non-targeting guides were selected from a previously derived list (2). Dual direct capture seq was performed as described previously(3). Briefly, CRA and CRI cell lines were infected with two pooled lentiviral libraries (one library for CRA, one for CRI) of 200 dual sgRNA vectors and three dual non-targeting vectors (6 non-targeting guides total). These cells were selected for purity with puromycin (2µg/mL), recovered for two days, and processed according to the protocol for Chromium Next GEM Single Cell 5' Library v1.1. All scRNA-seq libraries for WT and Perturb-seq were sequenced on a NovaSeq 6000.

Immunofluorescence

For all microscopy experiments, on day one, cells were plated in 8 well cell culture treated microscopy slides (ibidi, 80841) so the cells would be at about 70% confluency the next day. For immunofluorescence, on day two, cells were first fixed (4% paraformaldehyde in PBS) for 10 minutes, washed with PBS, and then blocked/permeabilized (2% Bovine Serum Albumin/0.1% Triton X in PBS) for one hour at room temperature. The wells were washed with PBS, and then primary antibodies were added (buffer: 0.5% Bovine Serum Albumin/0.1% Triton X in PBS) for one hour at room temperature. The wells were washed with PBS. Then, secondary antibodies, Hoechst 33342 (Thermo Scientific, 62249), and Alexa Fluor™ 546 Phalloidin (Invitrogen, A22283) were added to the wells and incubated for one hour at room temperature in the dark.

Finally, the wells were washed with PBS and imaged in PBS. For live-cell imaging of lysosomes and mitochondrial membrane potential, cells were plated as described above. On day two, manufacturer protocols were followed for both TMRE-Mitochondrial membrane potential staining (Abcam, ab113852) and lysosome LysoTracker™ Red DND-99 staining (Invitrogen, L7528). All microscopy quantification was done using ImageJ.

Beta-galactosidase staining

Manufacturer protocols were followed for the Senescence β-Galactosidase Staining (Cell Signaling Technology, 9860). To avoid evaporation of the β-Galactosidase stain overnight, which would lead to salt crystals precipitating out of solution, the slides were placed in a plastic container with water-soaked paper towels to create a humidity chamber. Quantification was done using ImageJ.

Proteasome activity

Manufacturer protocols were followed for the proteasome activity assay (Proteasome-Glo Chymotrypsin-like cell-based assay, Promega, G8660). The fluorescence was measured on the Promega GloMax plate reader.

Relative telomere length

Genomic DNA was extracted from approximately one million cells per condition (QIAamp DNA Blood Mini Kit, 51104). Relative telomere length was measured by quantitative polymerase chain reaction (qPCR), expressed as the ratio of telomere to single-copy gene abundance (T/S ratio) (4, 5). The inter assay coefficient of variation (CV) for this study is 2.7% ± 1.7%. The intraclass correlation (ICC) of duplicate DNA extraction from similar samples is 0.955 (CI: 0.914-0.977).

Methylation clock

Genomic DNA was extracted from approximately one million cells per condition (QIAamp DNA Blood Mini Kit, 51104). The genomic DNA was then brought to the Stanford Genomics Facility, where bisulfite conversion and methylation chip experiments using the Infinium MethylationEPIC Kit were conducted. For quantification of the methylation data, the methylclock package(6) was used. There were technical replicates for the control samples (CRA NT, CRI NT). Due to a small fraction of CpG islands having inconsistent methylation rates between repeats, these repeats had about 15 - 30 % variability in methylation clock results. To correct for this technical variation, for each CpG island, the difference of the repeats was divided by the mean of the repeats, and only those CpGs with less than 15 % variability were kept. The more variable CpGs were filtered out, meaning they did not contribute to the methylation clock calculations. The specific CpGs filtered out for CRA NT were also filtered out for CRA EZH2 and CRA E2F3; the CpGs filtered out for CRI NT were also filtered out for CRI STAT3 and CRI ZFX. Then, the mean value for the technical repeats was calculated.

Single-cell RNA Sequencing (scRNA-seq) Analysis

10x Genomics Cell Ranger and Scanpy (7) were computational packages used to analyze scRNA-seq data. The potential rejuvenation effect of the TF perturbations was measured by how well the gene expression profile in perturbed cells mimicked the gene expression profile in the early passage cells, compared to the late passage cells. We first computed the gene expression fold changes (log2) in the late passage cells compared to the early passage cells. Then, for each TF perturbation (CRA or CRI) we computed the gene expression fold changes (log2) by comparing the cells with the guides targeting the TF and the cells with the non-targeting guides (NT). We then computed the Pearson correlation of the gene expression changes between late passage and early passage cells and those between CRA or CRI targeted cells and the NT control (R_rej). The TF perturbations with the strongest negative R_rej had the most significant change in gene expression towards being like earlier passage cells.

Differentially expressed TF module analysis for selecting initial TF candidates for the screen

Briefly, the promoter region around the transcription start site of every gene was scanned with known TF motifs with their positional weight matrices. A motif score was calculated by scanning 5000 base pairs upstream from the transcription start site for each gene (8) and transformed into a Z score using the mean and standard deviation of the motif score for all the genes. To create a TF module, we selected all genes for a TF with a Z-score of at least 2.5; for TFs with fewer than 50 genes passing this cutoff, the top 50 genes were selected. To identify TFs related to gene expression differences between early and late passage cells, we performed a Welch's t-test on the log2 fold change between early and late passage cells for the genes in each TF module against all other genes.

Transcription Factor Module Analysis with SCENIC

To find the downstream transcriptional signatures of the TF perturbations, we performed TF module analysis using the SCENIC pipeline (SCENIC) (9, 10). Briefly, TF targets were inferred from the scRNA-seq data based on the co-variation between a given TF and a gene and the occurrence of the TF binding site motif in the promoter of the gene. A module activity score (AUCell score) was then computed for each module in each single cell. We then compared the AUCell scores from the perturbed cells (CRA or CRI) with the corresponding NT cells using a ranksum test to derive an AUC module score for the differential gene expression between the TF-perturbed and the NT cells. Similar calculations were done for WT young versus old cells. For TF module analysis of the mouse parabiosis data, mouse TF modules were obtained through ortholog mapping of genes in the human TF modules.

Statistical analysis for cellular assays

Experiments were conducted in at least three biological replicates for all cell assays for TF perturbations and NT control. For each biological replicate, a log fold change is calculated for the mean values (across single cells) for TF perturbed cells and NT control cells. P values were calculated using two sample T test comparing the log fold changes of different biological replicates to zero. * p values < 0.05, ** p < 0.01, and *** p values < 0.001.

Gene lists for the cluster heatmaps

To create the cluster heatmaps, a collection of sources was used to generate the gene lists. For proteasome genes, all human proteasome genes were included. For mitochondria and metabolism related genes, all mitochondrial genes (except those encoding tRNA) and the genes derived from the KEGG pathway for “KEGG_CITRATE_CYCLE_TCA_CYCLE”, ID M3985 were included. For the cluster heatmaps on TERT, SV40, and RAS cancer expression, the gene list came from Danielsson et al. (11). For genes commonly differentially expressed in cancer, the gene list came from Xu et al. (12). The log 2 fold change for every gene in each sub-list was found for WT passaged cells and TF perturbations, with no p value cut off. Then, fold changes for the genes were clustered using Euclidean distance as the distance metric.

Animals

In this study, 3-month-old male C57BL/6J mice from The Jackson Laboratory and 20-month-old male mice from the National Institutes of Aging were utilized. The mice were maintained under specific pathogen-free conditions with a 12-hour light-dark cycle. All procedures involving animal handling and care were conducted in accordance with the institutional guidelines approved by the Institutional Animal Care and Use Committee (IACUC) at the University of California, San Francisco.

EZH2 and EGFP overexpression with AAV8 lentivirus

To overexpress a gene of interest specifically in the liver, EZH2 or EGFP were cloned into a pAAV-TBG-ER-TurboID plasmid (Addgene, #149415) where TurboID was replaced, under the control of a thyroxine binding globulin (TBG) promoter. The plasmids were packaged into AAV8, which targets the liver, by PackGene. Mice were administered 2 x 10^11 viral genomes per mouse, a standard dosage for AAV8-based delivery.

Histology

For histologic evaluation, the left liver lobe was excised, sectioned into 3-5 fragments, each approximately 5 mm, and fixed overnight at 4°C in 10% Zinc-formalin (Anatech Ltd, 170) with agitation. The fragments were subsequently washed to remove Zinc-formalin and then submerged in 30% sucrose-PBS overnight at 4°C with agitation. One of the fragments was then embedded in O.C.T. Compound (Sakura Finetek USA, 4583), and 10-μm thick sections were prepared using a Thermo Fisher Scientific Microm HM 525 cryostat.

For visualization of general morphology, Hematoxylin and Eosin (H & E) Staining was conducted according to the manufacturer’s protocol for Hematoxylin and Eosin. Initially, sections were washed under tap water to remove O.C.T. for 2 minutes, then rinsed in distilled water. Nuclei staining was carried out using Mayer’s Hematoxylin (EMS, 26609-03) for 8 minutes, followed by a 10-minute rinse under tap water and additional rinses in distilled water and 95% ethanol. Counterstaining was performed with Eosin Y solution (Sigma-Aldrich, HT110116-500) for 1 minute. Before mounting with Permount xylene-based mounting medium (Fisher Chemical, SP15-500), the slides underwent dehydration through one change of 95% ethanol and two changes of 100% ethanol, each for 1 minute, and were cleared in two changes of xylene, each lasting 1 minute.

To examine collagen fibers and assess collagen deposition histologically, 10-μm thick liver sections were first washed for 2 minutes to remove O.C.T. compound and then rinsed in distilled water. The slides were mordanted in pre-heated Bouin's Fixative (EMS, 26367-01) at 56°C for 1 hour, followed by a gentle 5-minute wash under running tap water to eliminate picric acid. Staining was conducted using Weigert's Iron Hematoxylin working solution, prepared by mixing Weigert's Hematoxylin A (EMS, 26367-02) and Weigert's Hematoxylin B (EMS, 26367-03) in a 1:1 ratio. After staining, the slides were washed again under running tap water for 5 minutes and rinsed in distilled water. This was followed by staining with 1% Biebrich Scarlett-Acid Fuchsin solution (EMS, 26367-04) for 5 minutes and subsequent rinsing in distilled water through three changes. Slides were then transferred to 1% acetic acid for 1 minute, rinsed again in distilled water, dehydrated in 95% ethanol for 1 minute, followed by 100% ethanol for another minute, and cleared in xylene for 1 minute. Finally, the sections were mounted using Permount xylene-based mounting medium.

To assess lipid content and its histological accumulation, sections were initially washed in running tap water for 2 minutes to remove O.C.T. compound, followed by a rinse in distilled water. Sections were then stained with 0.5 %Oil Red O (EMS, 26609-01), which was pre-filtered through a 0.45 μm filter, for 15 minutes. Differentiation was performed in 85% propylene glycol (EMS, 26609-02) for 1 minute. This was followed by a 3-minute wash in running distilled water. Nuclei were counterstained with Mayer's hematoxylin for 1 minute, then the sections were washed briefly under running tap water, blued for 1 minute in 0.1% sodium bicarbonate solution, and washed again in running tap water for 2 minutes. Finally, sections were mounted using glycerin jelly (EMS, 17998-10).

Western Blot

Fifty milligrams of liver tissue were homogenized using RIPA buffer (150 mM sodium chloride, 1% Triton X-100, 0.5% sodium deoxycholate, 0.1% sodium dodecyl sulfate, and 50 mM Tris, pH 8.0). This buffer was supplemented with complete, EDTA-free protease inhibitor cocktail (Roche, 11873580001) and Halt phosphatase inhibitor cocktail (Thermo Fischer Scientific, 78420). Homogenization was performed using 1.4 mm ceramic bulk beads (Omni, 19-645) in an Omni Bead Ruptor Elite. The protein concentrations in the lysates were determined using the BCA Protein Assay Kit (Thermo Fischer Scientific, PI23225), adhering to the manufacturer's guidelines. Fifty micrograms of each lysate were separated by electrophoresis on 4-12% Criterion XT Bis-Tris protein gels (BioRad, 3450124) and transferred to membranes using Trans-Blot Turbo Midi 0.2 μm PVDF Transfer Packs (BioRad, 1704157) with the BioRad Trans-Blot Turbo Transfer System.

The membranes were washed with TBS (150 mM sodium chloride, 10 mM Tris-HCl, pH 7.4) and blocked with 5% skim milk (RPI, M17200-1000) in TBST (0.1% Tween 20 in TBS) for one hour. They were then incubated overnight at 4°C with primary antibodies at a 1:1000 dilution, specifically targeting EZH2 (CST, 5246S), p-SAPK/JNK (CST, 4668S), SAPK/JNK (CST, 9252S), EGFP (CST, 2956S), and p16INK4a (CST, 2971S). GAPDH (Abcam, ab8245) was used as a loading control at a 1:5000 dilution, also incubated overnight. Following incubation, the membranes were washed, and the primary antibodies were detected using a 1:5000 dilution of HRP-conjugated horse anti-mouse IgG (CST, 7076S) or goat anti-rabbit IgG (CST, 7074S). Protein bands were visualized using Clarity Western ECL substrate (BioRad, 1705060) for GAPDH and Clarity Max Western ECL substrate (BioRad, 1705062) for other targets, with images captured on a BioRad ChemiDoc MP imaging system.

Detailed list of materials

Detailed list of materials used is shown in Dataset S8.

Supplementary Figures

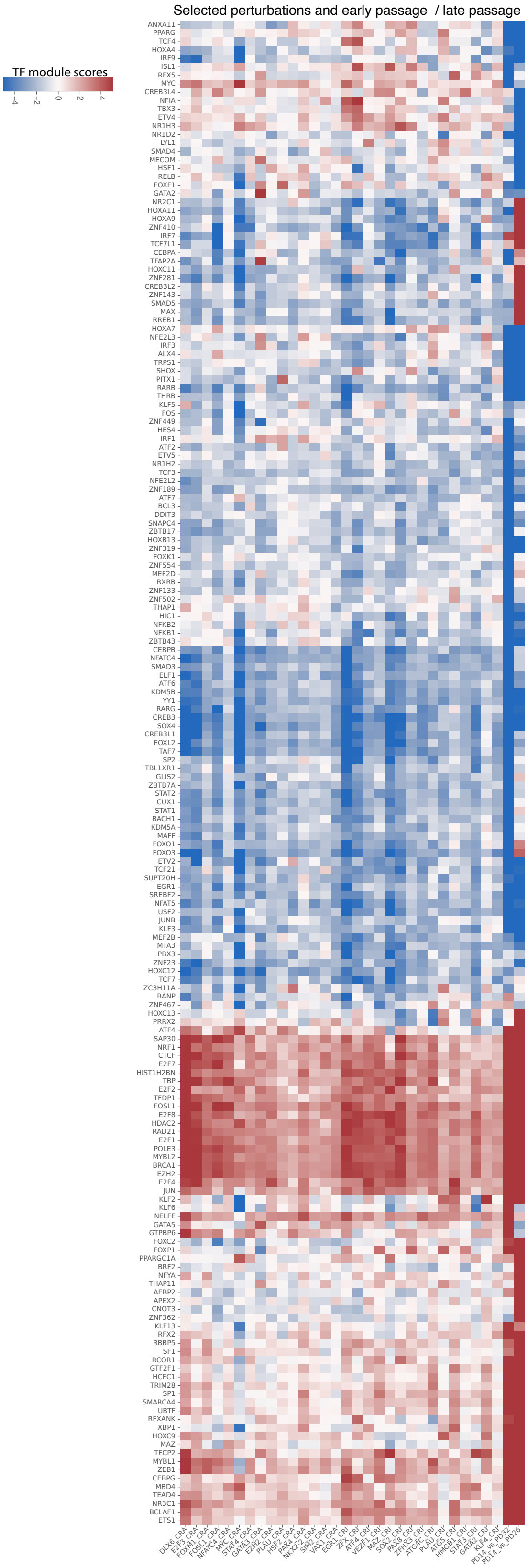
Selected perturbations and early passage / late passage
TF module scores
-4
-2
0
2
4
ANXA11
PPARG
TCF4
HOXA4
IRF9
ISL1
RFX5
MYC
CREB3L4
NFIA
TBX3
ETV4
NR1H3
NR1D2
LYL1
SMAD4
MECOM
HSF1
RELB
FOXF1
GATA2
NR2C1
HOXA11
HOXA9
ZNF410
IRF7
TCF7L1
CEBPA
TFAP2A
HOXC11
ZNF281
CREB3L2
ZNF143
SMAD5
MAX
RREB1
HOXA7
NFE2L3
IRF3
ALX4
TRPS1
SHOX
PITX1
RARB
THRB
KLF5
FOS
ZNF449
HES4
IRF1
ATF2
ETV5
NR1H2
TCF3
NFE2L2
ZNF189
ATF7
BCL3
DDIT3
SNAPC4
ZBTB17
HOXB13
ZNF319
FOXK1
ZNF554
MEF2D
RXRB
ZNF133
ZNF502
THAP1
HIC1
NFKB2
NFKB1
ZBTB43
CEBPB
NFATC4
SMAD3
ELF1
ATF6
KDM5B
YY1
RARG
CREB3
SOX4
CREB3L1
FOXL2
TAF7
SP2
TBL1XR1
GLIS2
ZBTB7A
STAT2
CUX1
STAT1
BACH1
KDM5A
MAFF
FOXO1
FOXO3
ETV2
TCF21
SUPT20H
EGR1
SREBF2
NFAT5
USF2
JUNB
KLF3
MEF2B
MTA3
PBX3
ZNF23
HOXC12
TCF7
ZC3H11A
BANP
ZNF467
HOXC13
PRRX2
ATF4
SAP30
NRF1
CTCF
E2F7
HIST1H2BN
TBP
E2F2
TFDP1
FOSL1
E2F8
HDAC2
RAD21
E2F1
POLE3
MYBL2
BRCA1
EZH2
E2F4
JUN
KLF2
KLF6
NELFE
GATA5
GTPBP6
FOXC2
FOXP1
PPARGC1A
BRF2
NFYA
THAP11
AEBP2
APEX2
CNOT3
ZNF362
KLF13
RFX2
RBBP5
SF1
RCOR1
GTF2F1
HCFC1
TRIM28
SP1
SMARCA4
UBTF
RFXANK
XBP1
HOXC9
MAZ
TFCP2
MYBL1
ZEB1
CEBPG
MBD4
TEAD4
NR3C1
BCLAF1
ETS1
DLX6_CRA
E2F3_CRA
FOXM1_CRA
FOSL1_CRA
NFATC4_CRA
MYC_CRA
STAT4_CRA
GATA3_CRA
EZH2_CRA
PLAU_CRA
HSF2_CRA
PAX4_CRA
NKX2-2_CRA
SIM2_CRA
VAX1_CRA
EGR1_CRI
ZFX_CRI
ATF4_CRI
VEZF1_CRI
MAZ_CRI
SOX2_CRI
PAX8_CRI
ZFHX3_CRI
ATG4C_CRI
PLAU_CRI
ATG5_CRI
HMGB1_CRI
STAT3_CRI
GATA2_CRI
KLF4_CRI
PD14_vs_PD32
PD14_vs_PD26

Figure S1. Transcription factor module analysis for selected TF perturbations (columns, Table1). Perturbations were clustered by the TF module scores (indicated by the color of the pixels) for TF modules (rows) from the SCENIC analysis. The TF module score is derived from the comparison of the AUCell scores between two groups of cells (see Methods). Modules differentially expressed in at least one pairwise comparison (|TF module score| > 5) were shown.

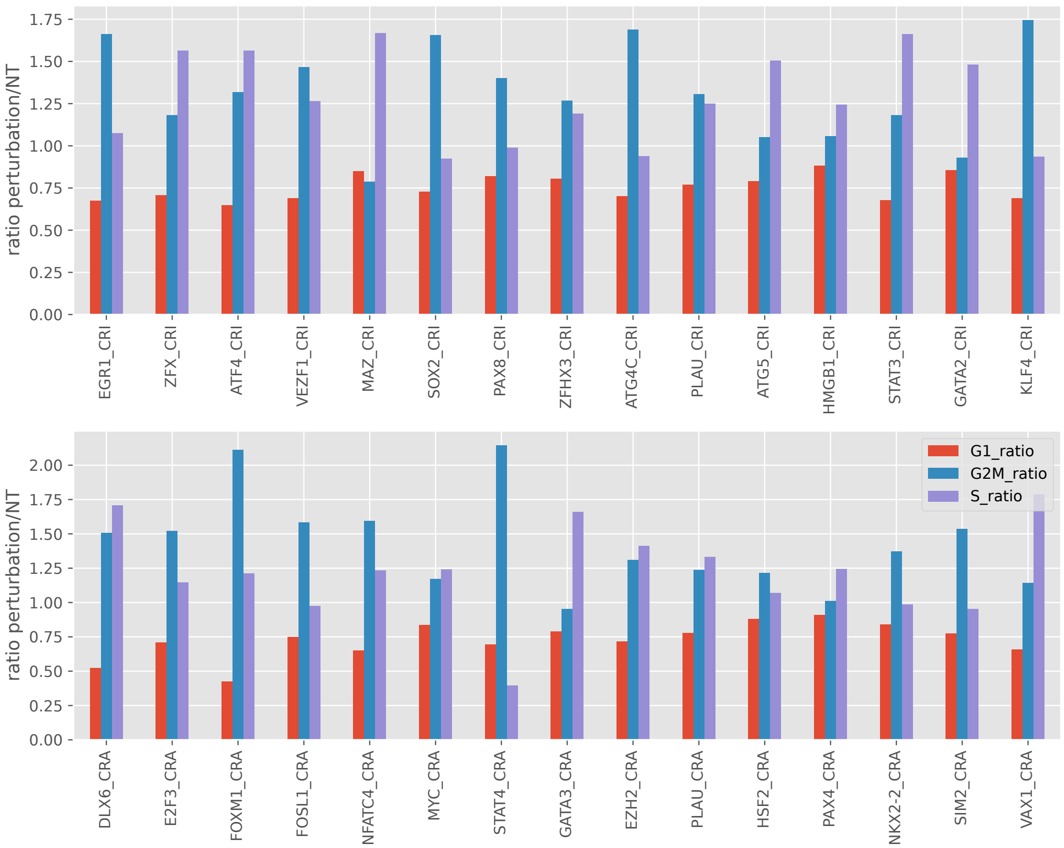

Figure S2. distribution of cells in different cell cycle stages for the top hit TFs from the CRIPSR_i (top panel) and CRISPR_a (bottom panel) screens. Percent of cells in a particular cell cycle stage is normalized to that for the NT cells.

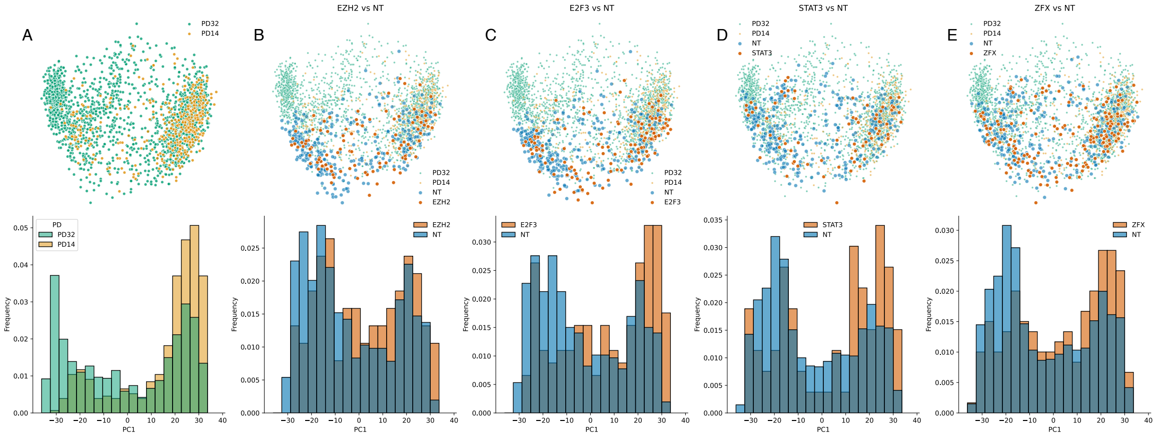

Figure S3. PCA plots of single cells with TF perturbations (Overexpression of EZH2 or E2F3, or repression of ZFX or STAT3) and NT control with reference to early and late passaged cells. A. PCA plot (Top panel) of early (PD=14) and late (PD=32) passaged cells. Bottom panel is the distribution when projected to the first principal component axis (PC1). **B to E.** PCA plots and distribution of cells on PC1 for overexpression of EZH2, E2F3, and repression of STAT3, ZFX. All four perturbations significantly shifted cell distribution towards that of younger cells.

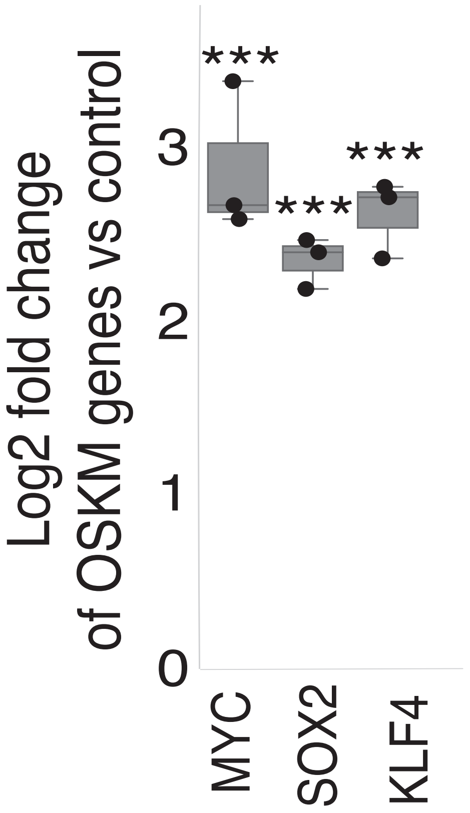

Figure S4. Quantitative PCR (qPCR) of cells overexpressing the Yamanaka factors. Late passage fibroblasts were treated with a plasmid for overexpressing the Yamanaka factors (OCT4, SOX2, KLF4, MYC) or a control plasmid. The log2 fold change is comparing the relative gene expression of those genes in the Yamanaka factor treated versus control cells. OCT4 was not expressed highly enough in control cells for quantification.

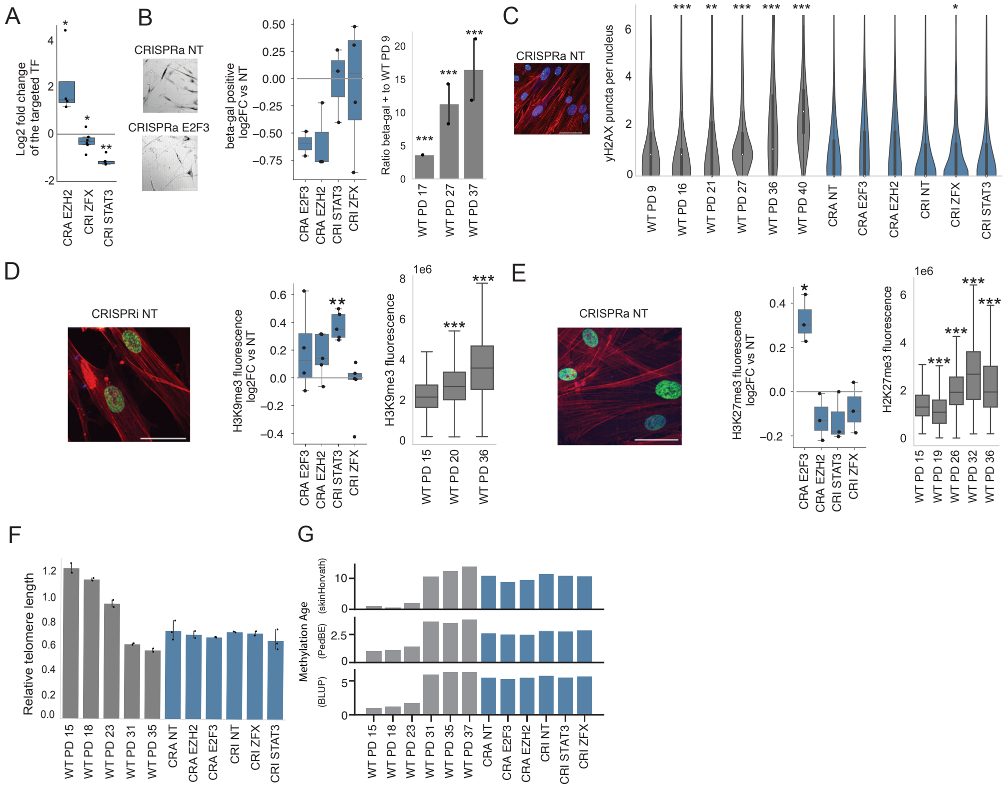

Figure S5. Further in vitro testing of the top four TF perturbations: CRISPRa E2F3, CRISPRa EZH2, CRISPRi STAT3, and CRISPRi ZFX. A. Quantitative PCR (qPCR) of the TF itself within CRISPRa EZH2, CRISPRi STAT3, and CRISPRi ZFX. CRISPRa E2F3 did not have significantly higher expression of E2F3 itself at the mRNA level but E2F3 target genes are significantly induced. **B.** Ratio of beta-galactosidase positive cells in the late passage CRISPRa and CRISPRi cells relative to their non-targeting control and the ratio in WT passaged fibroblasts relative to WT PD 9 (early passage). **C.** γH2AX DNA damage foci (puncta) per nucleus. **D.** H3K9me3 fluorescence per nucleus. **E.** H3K27me3 fluorescence per nucleus. **F.** Relative telomere length, as determined through qPCR analysis. **G.** Methylation clock assessment of WT passaged and CRISPRa/CRISPRi fibroblasts. Three different methylation clocks yielded similar trends. Methylation ages were normalized to that of the WT PD15 cells. 50 uM scale bar for all microscopy, * $p < 0.05$, ** $p < 0.01$, *** $p < 0.001$.

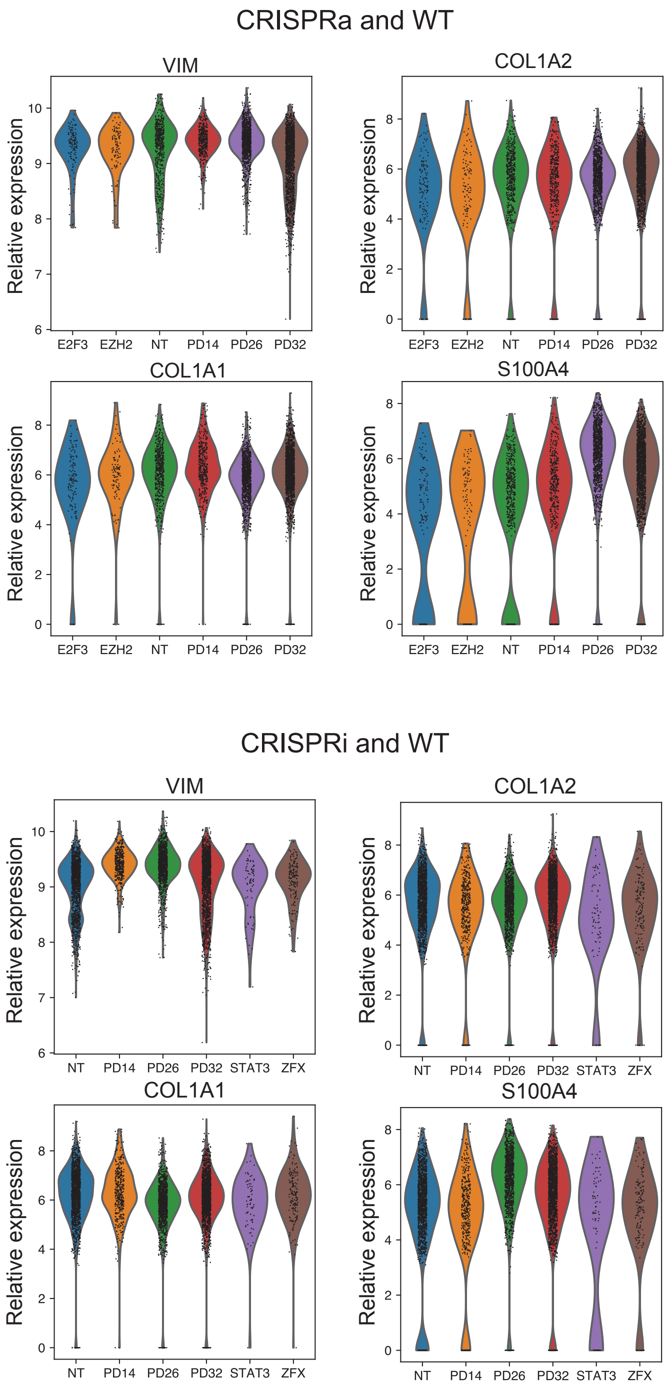

Figure S6. No signs of de-differentiation were seen across fibroblast marker genes in any treated or passaged fibroblasts. Single-cell RNA sequencing gene expression analysis was performed on the CRISPRa EZH2, CRISPRa E2F3, CRISPRi STAT3, CRISPRi ZFX, and passaged fibroblasts. Fibroblast marker genes including VIM, COL1A1, COL1A2, and S100A4 were assessed, and no differences were found.

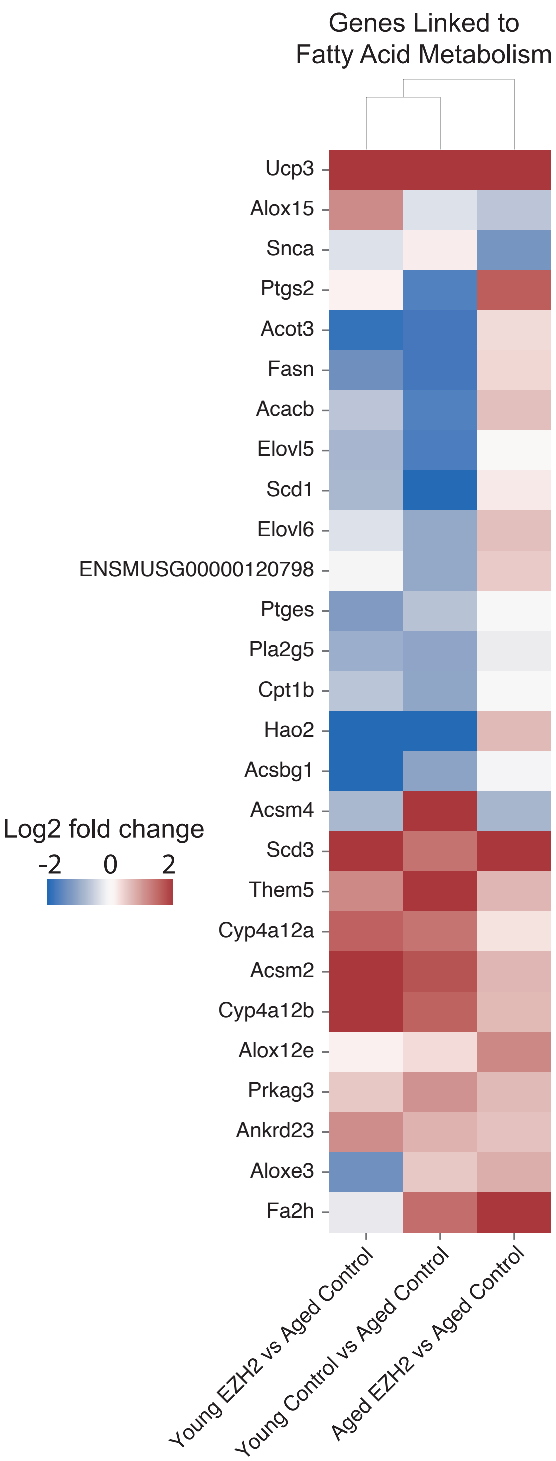

Figure S7. Lipid metabolism related genes returned to levels indicative of a more youthful state following EZH2 overexpression in old mouse livers. Bulk RNA sequencing was performed on young control, young EZH2, aged control, and aged EZH2 mice. Shown are gene expression changes (indicated by the color of the pixels) of lipid-metabolism related genes in three different pair-wise comparisons. Only genes with |log2(fold change)| > 1 in at least one pairwise comparison are shown.

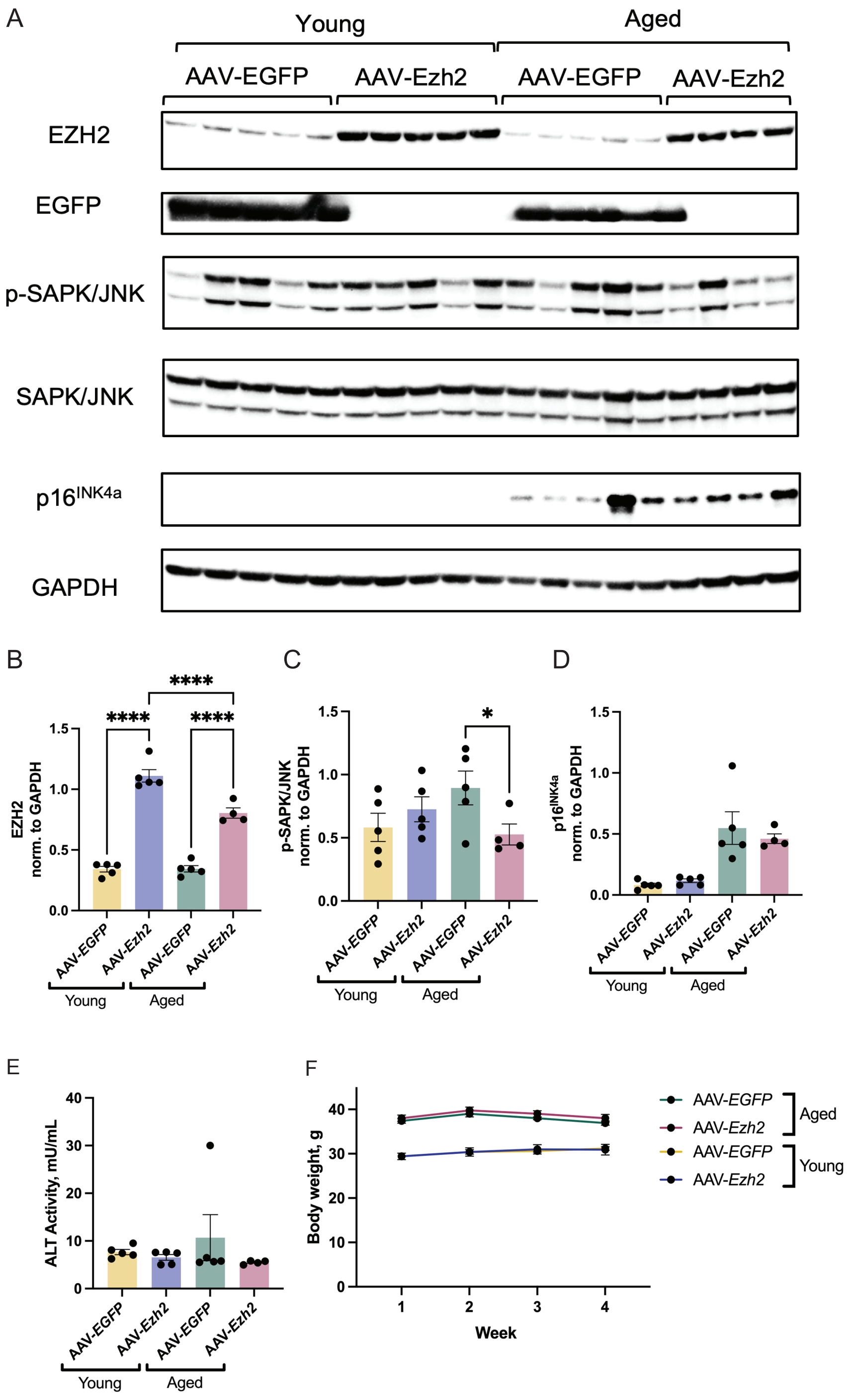

Figure S8. Protein quantification of changes in the livers of young and aged mice treated with either EGFP or EZH2 AAV8 overexpression. A. Livers from young and aged mice infected with either AAV-*EGFP* or AAV-*Ezh2* were lysed and subjected to western blotting with EZH2, EGFP, p-SAPK/JNK, SAPK/JNK, p16INK4a and GAPDH antibodies (n=4-5 per group). **B**. Quantification of WB analysis for EZH2 normalized to GAPDH. **C**. Quantification of WB analysis for p-SAPK/JNK normalized to GAPDH. **D**. Quantification of WB analysis for p16INK4a normalized to GAPDH. **E**. Quantification of ALT in sera from young and aged mice infected with either AAV-*EGFP* or AAV-*Ezh2*. **F**. Body weight of young and aged mice infected with either

AAV-*EGFP* or AAV-*Ezh2* over the course of the four-week experiment. Data are mean ± SEM. * $p < 0.05$, **** $p < 0.0001$ (one-way ANOVA with Šídák's correction for multiple comparison).

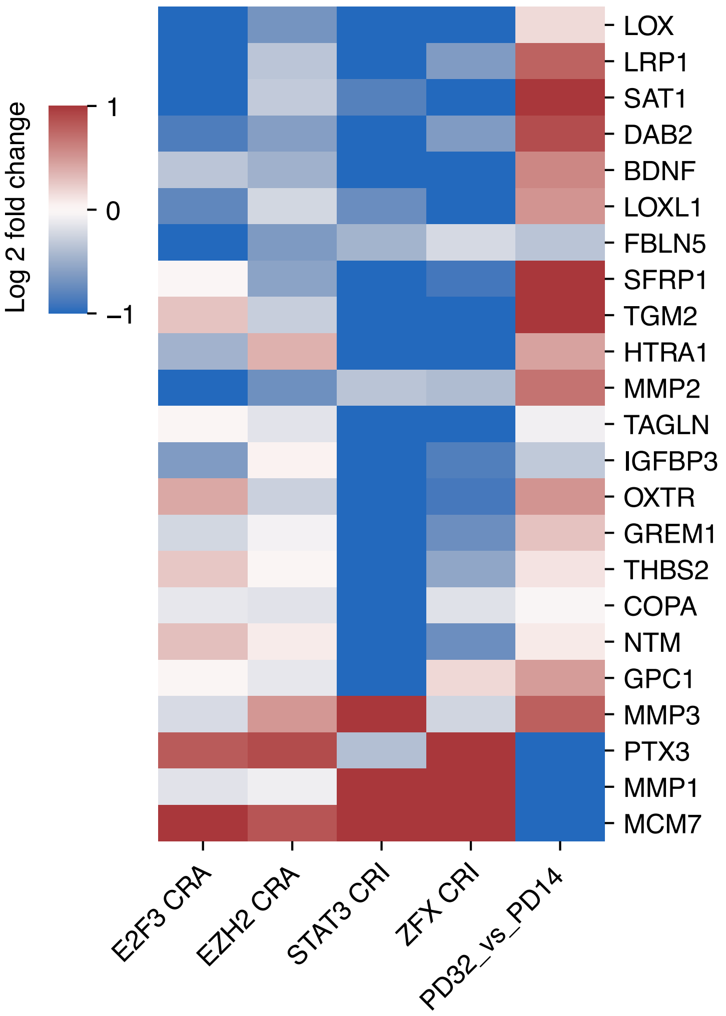

Figure S9. TF perturbations partially reverse "Mesenchymal Drift" (MD). A list of MD signature genes were taken from reference (13). A subset of these genes that changed (|log2fc| >1) in at least one of the perturbations were shown. Most of these genes are up-regulated in late passaged cells relative to the early passaged cells, exhibiting MD drift, while TF perturbations reversed the trend.

Dataset S1. List of 200 transcription factors tested.

Dataset S2. R_rej (and corresponding p-value calculated) for subsets of cells in different cell cycle phases. The column “All_corr” is the R_rej calculated when cells from all cell cycle phases are combined.

Dataset S3. Gene ontology (GO) enrichment analysis of genes down-regulated in late passaged cells (log2(fold change) > 0.5) but reversed with EZH2 over-expression (log(fold change) < -0.5). The analysis was restricted to a subset of cells classified as in G1 phase. GO terms with more than 500 genes (non-specific) were filtered out.

Dataset S4. Comparison of the R_rej from the perturbations in two opposite directions on the same transcription factors.

Dataset S5. Genes differentially expressed in old mouse liver relative to young mouse liver but reversed by EZH2 over-expression, and their GO enrichment analysis.

Dataset S6. Genes up/down regulated across mouse liver cancer models and their GO enrichment analysis.

Dataset S7. List of qPCR primers used.

Dataset S8. Detailed list of materials used.

SI References

1. C. S. McGinnis, *et al.*, MULTI-seq: sample multiplexing for single-cell RNA sequencing using lipid-tagged indices. *Nat. Methods* **16**, 619–626 (2019).

2. M. A. Horlbeck, *et al.*, Compact and highly active next-generation libraries for CRISPR-mediated gene repression and activation. *Elife* **5** (2016).

3. J. M. Replogle, *et al.*, Combinatorial single-cell CRISPR screens by direct guide RNA capture and targeted sequencing. *Nat. Biotechnol.* **38**, 954–961 (2020).

4. R. M. Cawthon, Telomere measurement by quantitative PCR. *Nucleic Acids Res.* **30**, e47 (2002).

5. J. Lin, *et al.*, Analyses and comparisons of telomerase activity and telomere length in human T and B cells: insights for epidemiology of telomere maintenance. *J. Immunol. Methods* **352**, 71–80 (2010).

6. D. Pelegí-Sisó, P. de Prado, J. Ronkainen, M. Bustamante, J. R. González, methylclock: a Bioconductor package to estimate DNA methylation age. *Bioinformatics* **37**, 1759–1760 (2021).

7. F. A. Wolf, P. Angerer, F. J. Theis, SCANPY: large-scale single-cell gene expression data analysis. *Genome Biol.* **19** (2018).

8. E. Lee, H. J. Bussemaker, Identifying the genetic determinants of transcription factor activity. *Mol. Syst. Biol.* **6**, 412 (2010).

9. B. Van de Sande, *et al.*, A scalable SCENIC workflow for single-cell gene regulatory network analysis. *Nat. Protoc.* **15**, 2247–2276 (2020).

10. S. Aibar, *et al.*, SCENIC: single-cell regulatory network inference and clustering. *Nat. Methods* **14**, 1083–1086 (2017).

11. F. Danielsson, *et al.*, Majority of differentially expressed genes are down-regulated during malignant transformation in a four-stage model. *Proc. Natl. Acad. Sci. U. S. A.* **110**, 6853–6858 (2013).

12. K. Xu, *et al.*, A comparative analysis of gene-expression data of multiple cancer types. *PLoS One* **5**, e13696 (2010).

13. J. Y. Lu, *et al.*, Prevalent mesenchymal drift in aging and disease is reversed by partial reprogramming. *Cell* **188**, 5895-5911.e17 (2025).
